# T22. PITUITARY GLAND VOLUME DIFFERENCES IN INDIVIDUALS WITH PSYCHOSIS: RESULTS FROM THE BIPOLAR-SCHIZOPHRENIA NETWORK ON INTERMEDIATE PHENOTYPES (B-SNIP) STUDY

**DOI:** 10.1093/schbul/sby016.298

**Published:** 2018-04-01

**Authors:** Synthia Guimond, Samantha Tingue, Gabriel A Devenyi, Yun-Xiang Tang, Luke Mike, M Mallar Chakravarty, John A Sweeney, Godfrey D Pearlson, Brett A Clementz, Carol A Tamminga, Matcheri S Keshavan

**Affiliations:** 1 Harvard Medical School, Beth Israel Deaconess Medical Center; 2 Emmanuel College, Beth Israel Deaconess Center; 3 McGill University; 4 Second Military Medical University; 5 Beth Israel Deaconess Center; 6 University of Texas Southwestern; 7 Yale University; 8 University of Georgia; 9 University of Texas Southwestern Medical Center

## Abstract

**Background:**

When exposed to stress, the hypothalamic-pituitary-adrenal axis is hyperactivated, which can cause the enlargement of the pituitary gland. Hence, pituitary gland volume could be a biomarker of stress present in psychosis. However, it remains unclear if individuals with psychosis have larger pituitary gland than healthy people. Previous studies investigating this question used small samples and reported inconsistent results. In the current study, we used an automated multi-atlas segmentation method to investigate the differences between pituitary gland volumes in a large sample of individuals on the psychosis spectrum.

**Methods:**

Data collection was completed across six sites in the Bipolar-Schizophrenia Network on Intermediate Phenotypes (B-SNIP) consortium with a total of 755 participants included in the study - 174 individuals with schizophrenia (SZ), 115 with schizoaffective disorder (SZA), 167 with psychotic bipolar disorder (PBD), and 299 healthy controls (HC). Structural magnetic resonance images were acquired and pituitary gland volumes were obtained using the automated MAGeT-Brain algorithm. General linear model and post-hoc independent t-tests were used to analysis the differences between subgroups of patients using clinical diagnosis and agnostic Biotype classification (Biotype 1 being the most cognitively impaired). We also explored potential effect of antipsychotic intake, symptoms severity and duration of illness. In all analyses, we used Bonferroni correction for multiple comparisons and entered confounds as covariates (age, sex, race, intracranial volume, and site).

**Results:**

Overall, the pituitary gland volumes were not significantly different between patients and HC. No significant main effect of diagnosis was observed, but SZ patients had trending larger pituitary volume compared to HC (p=.033, uncorrected). We observed a significant main effect of Biotype (p=.003), with Biotype 1 having significantly larger pituitary gland than HC and Biotype 2 (p=.004 and p=.013). In the patients group, no significant relationship between the pituitary gland and the amount of antipsychotic intake was observed (r=.02, p=.68). Significant correlations with the pituitary gland volume were observed with symptoms severity (r=.22, p=.000), and with the duration of illness (r=-.18, p=.002). Importantly, Biotypes did not significantly differ in terms of symptoms severity nor duration of illness.

**Discussion:**

As a group, individuals with psychosis do not have abnormal pituitary gland volume, but larger pituitary gland is related to shorter duration of illness and greater symptoms severity. Therefore, larger pituitary gland volume could be a state-related biomarker of psychosis. Moreover, while we did not observe any significant subgroup differences using clinical diagnosis, our results suggest an increase in pituitary volume in biotype 1 patients compared to HC. These findings clarify previous inconsistent reports, and encourage further investigation of stress biomarkers in individual with psychosis with lower cognitive abilities. In the future, this could lead to the development of more targeted treatments for this specific subgroup of patients.

